# Quantitative assessment of thermal ablation in small animal tumor models: implications for pre-clinical studies of combination treatments

**DOI:** 10.1088/1361-6560/adfeb2

**Published:** 2025-09-04

**Authors:** Anna Bottiglieri, Punit Prakash

**Affiliations:** Department of Biomedical Engineering, The George Washington University, 5000 Science and Engineering Hall, Washington, DC 20052, United States of America

**Keywords:** radiofrequency ablation, thermal ablation, bioheat transfer modeling, imaging-based computational models, tumor biophysics characteristics, pre-clinical studies

## Abstract

*Background*. Radiofrequency (RF) and other thermal ablation modalities in combination with systemic therapies are the subject of investigations for the treatment of tumors at risk of recurrence after first line therapies. The potential of combined therapeutic approaches is studied largely in small animals where size constraints and tumor-specific physical characteristics may significantly influence the local heating. Spatial-temporal analyses of tissue temperature profiles (i.e, thermal dosimetry) are largely neglected in preclinical studies, thus limiting the reliable assessment and interpretation of biological outcomes. *Approach*. Imaging-based information and complementary experimental reports of the (1) heat transfer conditions on the external surface of the tumor, (2) RF applicator position, (3) tumor shape and dimension, and (4) blood flow in major intra-tumoral vessels acquired from a total of *N* = 15 rat tumor models were used to build computational models of a local RF-hyperthermia system within a subcutaneous tumor. Analysis of the influence of each variable on 3D temperature profiles and on the percentage of tumor volume above lethal (50 °C) and below irreversible thermal damage (43 °C) thresholds, *V*_50_ and *V*_43_, were conducted at 15 min of coupled electromagnetic-thermal transfer simulations. *Results:* For each examined variable, changes in *V*_50_ values ranged between (1) 9%–31%, (2) 9%–18%, (3) 4%–58%, and (4) 12%–19% and those in *V*_43_ are between (1) 24%–74%, (2) 51%–78%, (3) 3%–86%, and (4) 58%–65% indicating sources of considerable variability in tissue thermal profiles. *Conclusions.* Information of ablation procedure parameters, i.e. estimates of the RF applicator location, and accessible measurements of tumor biophysical characteristics, i.e. tumor size, shape and blood flow profile in major arteries, should be consistently reported in experimental studies of local heating in small animals in order to study heat-induced bioeffects and compare the outcomes in combination approaches with systemic therapies.

## 1. Introduction

Thermal interventions such as radiofrequency (RF) and microwave (MW) ablation, and cryoablation are widely accepted minimally invasive techniques to treat solid tumors by locally increasing (RF and MW ablation) or decreasing (cryoablation) tissue temperature to induce cytotoxic effects on cancer cells (Chu and Dupuy 2014). Most recent guidelines (Reig *et al* 2022, NCCN Guidelines 2025) include RF and MW ablation as first-line treatment modalities for early-stage hepatocellular carcinoma (HCC) in patients who are not candidates for transplantation or surgical resection. Moreover, a recent phase 3 clinical trial demonstrated thermal ablation to be non-inferior to standard-of-care surgical resection for treatment of colorectal liver metastases (Van Der Lei *et al* 2025). In addition, thermal ablation techniques can be considered for the treatment of medically inoperable stage I and II non-small cell lung cancer (NCCN Guidelines 2025).

RF and MW ablation yield a temperature gradient in tissue: in the tumor region next to the RF applicator, temperatures exceed 50 °C–60 °C (Desai *et al* 2020) thus inducing tissue coagulation necrosis; following a radial decrease toward the periphery of the tumor, temperatures in the range of 40 °C–43 °C are observed between the area of coagulation necrosis and normal tissue. Sublethal bioeffects, which include increase of blood perfusion (hyperemia), transient decrease of intratumoral pressure and tumor stiffness, as well as augmented infiltration of innate immune cells and cytokines, are biophysical signatures of the peri-ablation zone (Chu and Dupuy 2014, Lemdani *et al* 2019, Dewhirst *et al* 2022, Muñoz *et al* 2022, Odéen *et al* 2023).

In light of these thermal-induced bioeffects, a growing body of studies, largely in the preclinical setting of small animals, are devoted to investigating the possible advantages and risks of local thermal interventions in combination with systemic therapies (Slovak *et al* 2017, Qi *et al* 2020, Kan *et al* 2022, Santana *et al* 2023). These studies are motivated by the clinical need to identify therapeutic schemes to treat tumors at risk of recurrence after first line treatments (Qin *et al* 2023). Determining whether and in which patient early use of systemic therapy in combination with thermal ablation yields survival benefit could represent a significant advance in the treatment options available to patients.

The current study presents an imaging-based computational modeling approach for intra-tumoral thermal dosimetry during RF ablation studies in pre-clinical small-animal experiments. The imaging-informed computational models provide estimates of the spatial temperature distribution in small animal tumor models—this information is relevant to interpret and assess any post-heating experimentally observed bioeffects.

Small animal tumor models are instrumental to identify the therapy sequencing that optimizes the tumor response to combined treatments (Obeid *et al* 2018), including those with local thermal therapies (Lemdani *et al* 2019, Tian *et al* 2022, Santana *et al* 2023, Seguin *et al* 2024). However, temperature profiles across experiments could considerably vary due to tumor-specific anatomical and physiological characteristics, even when consistent generator input power and ablation duration are used. Indeed, a number of factors including tumor size and shape, and blood perfusion can substantially impact temperature profiles induced in tumors. Variabilities in the thermal profile can initiate different biological and immunological mechanisms depending on the local temperature increase and the fraction of the tumor volume exposed to lethal and sub-lethal temperatures that ultimately will influence the response to the therapeutic approach under scrutiny (Dewhirst *et al* 2022).

Extant reports of preclinical studies show a widespread use of clinical ablation applicators, which are primarily designed to deliver heating in human tumors up to 4 cm diameter, in small animal tumor models which have maximal diameters rarely exceeding ∼10−15 mm (Obeid *et al* 2018, Muñoz *et al* 2020, Kan *et al* 2022, Moussa *et al* 2023). Because of the large diameter of clinical ablation applicators (∼14–17 gauge) relative to the size of the tumor, investigators are forced to apply local heating for a time significantly shorter (3 s–5 min) compared to the time of clinical procedures such as RF ablation (10–15 min). Furthermore, time-temperature profiles are often measured only at the RF applicator, thus relevant information about the temperature gradient within the tumor volume and peri-ablation zone are neglected (Markezana *et al* 2021, Muñoz *et al* 2022, Tian *et al* 2022, Santana *et al* 2023). Finally, analysis of variability in spatiotemporal or, at least, temporal profiles of the temperature among the experiments for a reliable interpretation of the heat-induced bioeffects is largely missing or under-reported.

Altogether, these factors can lead to pre-clinical thermal interventions inducing substantially different time-temperature profiles in tissue as compared to the clinical setting, as well as considerable variability in time-temperature profiles within treatment groups. These variabilities in thermal profiles can be anticipated to yield considerable variability in biological outcomes.

Thus, there is a need to (1) design experimental treatment protocols that are clinically relevant in terms of target temperature, time, and heating rate; (2) use systems for local delivery of heating that are scaled to fit small animal tumor models in order to sustain temperature profiles over durations similar to those delivered in clinical settings; and (3) quantitatively assess spatio-temporal temperature profiles incorporating tumor-and procedure-related information that are relevant to ensure reliable assessment of the biological and immunological effects after heating and reproducibility of the studies.

In a prior experimental study aimed at studying changes in intra-tumoral pressure and stiffness as a function of intensity of heating, we used a generic computational modeling approach (i.e. RF applicator perpendicularly inserted into an idealized tissue geometry) to design an RF heating system for delivering thermal interventions in a small animal tumor model of HCC for a time (15 min) representative of clinical RF ablation procedures (Bottiglieri *et al* 2025). In the present study, focused on need (3), we present an approach for incorporating tumor- and procedure-related information from peri-procedural ultrasound imaging acquisitions and experimental reports into computational models of thermal dosimetry.

The present study has been conceptualized as an experimentally-driven analysis of variability in the intra-tumoral thermal profiles due to: (1) heat transfer conditions between the external surface of the subcutaneous tumor model and the environment; (2) positioning of the RF-heating applicator within tumors; (3) size and shape of the tumors; and (4) blood flow characteristics within major intra-tumoral vessels. Up to date, the influence of those factors, particularly blood vessels and position of the RF applicator on the temperature profiles have been studied in the context of emulating clinical interventions and, to some extent, in the pre-clinical settings of large animals that typically do not present tumors. To the best of our knowledge, this is a first study presenting a framework where *in vivo* parameters extracted from ultrasound imaging and Doppler ultrasound have been used to refine bioheat transfer simulations of thermal doses delivered in small animal tumors with an approach recapitulating ultrasound-guided RF thermal interventions. This approach presents a means for investigators to employ computational models informed by widely-available imaging modalities (e.g. ultrasound imaging and Doppler ultrasound) to assess 3D spatio-temporal thermal profiles in pre-clinical settings, and thereby support interpretation of biological outcomes.

## 2. Methodology

### 2.1. Study design: identification of variables and computational modeling workflow

We previously reported on the development of an RF hyperthermia (RFHT) system, informed by a generic computational model, for delivering controlled local heating to subcutaneous tumors (Bottiglieri *et al* 2025). In the prior study, we employed the system to induce non-lethal (‘low RFHT’) and lethal temperatures (‘high RFHT’), respectively, in more than 50% of the tumor. The computational model employed in the previous study consisted of the RF applicator inserted perpendicular to the skin surface into a ∼12 mm diameter spherical tumor (‘reference scenario model’). This bioheat transfer computational model was used to guide selection of energy delivery settings to achieve low RFHT (hyperthermia temperatures) and high RFHT (ablative temperatures) thermal doses. Experiments were performed using a subcutaneous rat model of HCC in a total of 15 tumors across 8 rats (7 tumors for the low RFHT and 8 tumors for the high RFHT group). The simulated peak temperature values maintained during 15 min of RF energy delivery were 77 °C and 47 °C for high and low RFHT thermal doses, respectively. The temperature values measured at the tip of the customized RF applicator were 74.1 ± 5.2 °C and 45.9 ± 1.6 °C, thus showing agreement between computational and experimental temperature-time profiles.

**Figure 1.**
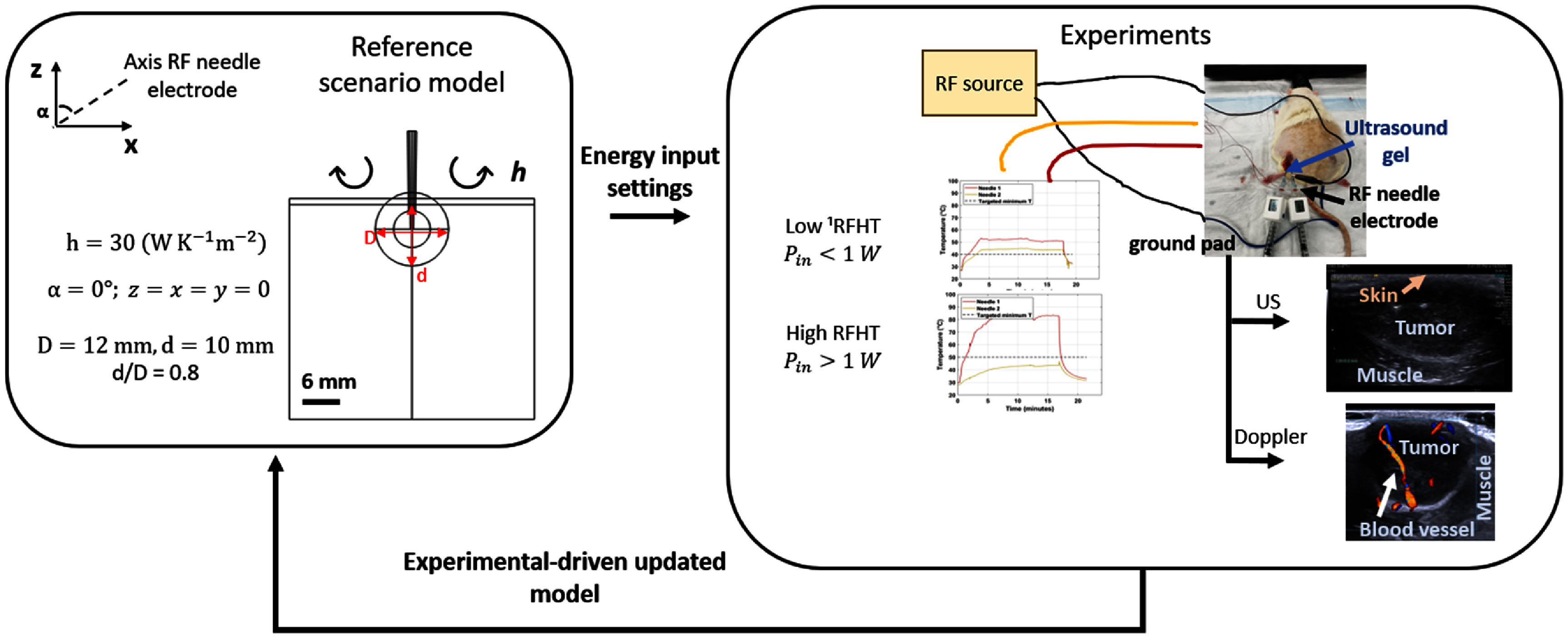
Schema of the workflow illustrating the synergy between the computational modeling study and experimental study to: (1) build a generic model (reference scenario) used to identify the energy input settings to deliver low and high RFHT in a subcutaneous rat tumor model of HCC; (2) build bioheat transfer computational models to quantify the influence of tumor- and procedure-related variables identified from ultrasound and Doppler ultrasound imaging on temperature spatial profiles. ^1^RFHT: radiofrequency hyperthermia.

During experiments, Doppler ultrasound imaging was employed to assess blood flow in major vessels prior to initiating interventions, and conventional B-mode ultrasound was used to assess tumor geometry and guide the placement of the RF-heating needle. Analysis of imaging data from these experiments indicated anatomic and physiological variations from the generic reference model, that we hypothesized may influence the resultant spatio-temporal temperature profiles. Figure 1 shows the workflow we implemented in the present study for comparative analysis of thermal profiles against those predicted by the generic computational model, that for the present investigation will serve as the reference scenario model.

The computational model geometry employed in the present study is as follows. The geometry of a 23-gauge needle with 4 mm exposed tip has been centrally positioned within a 12 mm diameter spherical tumor geometry placed in a cylinder (35 mm height × 40 mm diameter) modeling the surrounding muscle tissue and a 2 mm skin layer. A convective heat transfer coefficient *h* = 30 (${\text{W}}{{\text{m}}^{ - 3}}{{\text{K}}^{ - 1}}$) was assigned to the upper surface of the cylinder (‘skin’) to model the natural convection mechanisms between the external environment at *T* = 20 °C and the rat’s skin surface; *T* = 35 °C was used as the initial thermal condition for all geometrical domains. The input power was assigned as electrical boundary conditions to the proximal end of the RF applicator geometry. Power was applied in a manner to mimic the experimental procedure, with an initial ramp phase until needle temperature reached the setpoint temperature (77 °C for ‘high RFHT’; 47 °C for ‘low RFHT’), maintained for the remainder of the 15 min heating procedure. The input power to maintain the designed temperature at the tip of the RF applicator, i.e. 77 °C (high RFHT) and 47 °C (low RFHT) up to 15 min of RFHT, was on average 0.7 W (low RFHT) and 1.5 W (high RFHT). Zero voltage value was assigned to bottom of the cylinder to model the return electrode closing the RF electrical circuit. Electrical insulation boundary conditions were applied on all other external surfaces of the computational domain.

Computational simulations, implemented using the finite element method (COMSOL Multiphysics, v6.3, Burlington, MA) numerically solved Maxwell’s equations at 500 kHz assuming quasi-static conditions and coupled with the bioheat transfer equation (equation (1)).

\begin{equation*}\rho c{\text{ }}\frac{{\partial T}}{{\partial t}} = \nabla \cdot k\left( T \right)\nabla T + {\text{Q}}\left( {{T}} \right) - {c_{\text{bl}}}{m_{\text{bl}}}\left( {T - {T_{\text{bl}}}} \right).\end{equation*}

The term ${{Q}}\left( {{T}} \right) = {{\sigma }}\left( {{T}} \right){\text{ }}{\left| {\nabla V} \right|^2}$ describes the resistive heat losses within the tissue when RF electric current is applied. $\nabla {\text{ }}\cdot k\nabla T$ is the term describing the passive heating of tissue via thermal conduction. The term ${c_{\text{bl}}}{m_{\text{bl}}}\left( {T - {T_{\text{bl}}}} \right)$ describes the heat sink due to microvascular blood perfusion. Temperature-dependent models for thermal conductivity ($k$), electrical conductivity ($\sigma $) and blood perfusion (m_bl_) have been implemented as described in (Song *et al* 1984, Rossmann and Haemmerich 2014, Bottiglieri *et al* 2023). In particular, the electrical conductivity $\sigma \left( T \right)$ which is the driver for the dissipated electrical energy within the tissue, varies from 0.45 (S m^−1^) at body temperatures to 0.94 (S m^−1^) at *T* = 80 °C (Rossmann and Haemmerich 2014). Table 1 lists the baseline values of the electrical and thermal properties in equation (1) for both normal and tumor tissue.

**Table 1.** Baseline values of solid tissue (i.e. muscle, tumor, skin) and blood paramters used in the bioheat transfer equation (equation (1)) and modified bioheat transfer equation (equation (2)).

Property	Symbol (Unit)	Value (solid tissue type)	References
Thermal conductivity	$k\left( {{\text{W}}{{\text{m}}^{ - 1}}{{\text{K}}^{ - 1}}} \right)$	0.5, 0.6, 0.4 (muscle, tumor, skin)	Rossmann and Haemmerich (2014), Hasgall *et al* (2022)
Density solid tissue	$\rho \left( {{\text{kg}}{\text{m}^{ - 3}}} \right)$	1080 (muscle, tumor, skin)	Hasgall *et al* (2022)
Heat capacity solid tissue	$c\left( {{\text{J}}{{\text{K}}^{ - 1}}{\text{k}}{{\text{g}}^{ - 1}}} \right)$	3425 (muscle, tumor, skin)	Rossmann and Haemmerich (2014)
Electrical conductivity	$\sigma \left( {{\text{S}}{\text{m}^{ - 1}}} \right)$	0.45, 0.3, 0.005 (muscle, tumor, skin)	Hasgall *et al* (2022), Haemmerich *et al* (2003a)
Blood perfusion	${m_{\text{bl}}}\left( {{\text{kg}}{\text{m}^{ - 3}}{\text{s}^{ - 1}}} \right)$	0.515	Song *et al* (1984), Bottiglieri *et al* (2023)
Heat capacity blood	${c_{\text{bl}}}\left( {{\text{J}}{{\text{K}}^{ - 1}}{\text{k}}{{\text{g}}^{ - 1}}} \right)$	3617	Hasgall *et al* (2022)
Dynamic viscosity blood	$\mu \left( {{\text{kg}}{{\text{m}}^{ - 1}}{{\text{s}}^{ - 1}}} \right)$	2.1 × 10^−3^	González-Suárez *et al* (2018)
Density blood	${\rho _{\text{bl}}}\left( {{\text{kg}}{\text{m}^{ - 3}}} \right)$	1050	Hasgall *et al* (2022)

We observed relevant variations in the following variables across the experiments as compared to the assumptions made for the reference model scenario that could influence the predicted temperature profiles:

1. Thermal boundary conditions between skin and air
2. Position of the RF-applicator within the tumor
3. Maximum diameter and shape of the tumor
4. Blood flow characteristics in major intra-tumoral vessels, in addition to variables 1–3

Thus, the reference scenario model (figure 1) has been modified to account for the variables/factors (1) to (4). A total of 24 electromagnetic-thermal simulations were performed, 12 each for low and high RFHT, consistent with the thermal doses used in our previous experimental study (Bottiglieri *et al* 2025). All simulations were run considering a 15 min heating duration.

*1. Thermal boundary conditions between skin-ultrasound gel and air*

For all experimental procedures, we used an ultrasound imaging-guided approach to place the customized RF applicator in each subcutaneous tumor for both low and high RFHT treatment groups. The application of ultrasound gel on the surface of each tumor is required to ensure coupling of the acoustic waves transmitted from the ultrasound transducer into the tissue. In the reference scenario model that we previously developed (Bottiglieri *et al* 2025), we assumed a convective heat transfer coefficient of *h* = 30 (${\text{W}}{{\text{m}}^{ - 3}}{{\text{K}}^{ - 1}}$) to model the heat exchange between the skin of the animal and the air (Incropera *et al* 2007). While a convective heat transfer boundary condition may be suitable for skin surface exposed to ambient conditions, such a boundary condition may not be representative of the experimental conditions when ultrasound gel is applied on the skin surface for imaging acquisition, given that the gel typically has a viscosity ranging between 100–185 (${\text{Pa}}{\text{s}^{ - 1}}$, Technical Datasheet 2011), and a temperature ∼20 °C. Together, the high viscosity and relatively lower temperature of the gel (∼20 °C) as compared to the body peripheral temperature (∼30 °C–35 °C) could reduce the convective heat exchange between skin and air.

Temperature at the skin of the tumor cannot be reliably measured using non-invasive methods, such as thermal camera, during US-guided RF ablation procedures. For these reasons, we used computational models to formulate an hypothesis on the possible impact of the physical properties of the ultrasound gel, i.e. low temperature and high viscosity, on the heat-exchange convection mechanism at the surface of the tumor and assess the implication for the temperature rise in the tumor and adjacent tissue. To this end, we modified the initially assumed thermal boundary conditions at the skin-air interface, *h* = 30 (${\text{W}}{{\text{m}}^{ - 3}}{{\text{K}}^{ - 1}}$), to evaluate the independent effect on the temperature profile of:

• *h* = 15 (${\text{W}}{{\text{m}}^{ - 3}}{{\text{K}}^{ - 1}}$),
• *h* = 5 (${\text{W}}{{\text{m}}^{ - 3}}{{\text{K}}^{ - 1}}$),
• *T* = 30 °C (constant)

Where, *h* = 15 (${\text{W}}{{\text{m}}^{ - 3}}{{\text{K}}^{ - 1}}$) and *h* = 5 (${\text{W}}{{\text{m}}^{ - 3}}{{\text{K}}^{ - 1}}$) account for two different degrees of heat exchange by convection, both of them lower than the initial assumption; *T* = 30 °C (constant) accounts for both high viscosity and lower temperature of the ultrasound gel compared to the tissue that together could considerably inhibit the local increase of the temperature.

*2. Position of the RF-applicator within the tumor*

In the reference scenario model (figure 1), the position of the ablation applicator was assumed aligned perpendicular to the surface (*α* = 0°) of the tissue geometry and the active electrode length centrally placed within the tumor domain (*x* = *y* = *z* = 0). Ultrasound images that we examined revealed differences in the positioning of the needle as compared to the reference scenario model.

**Figure 2.**
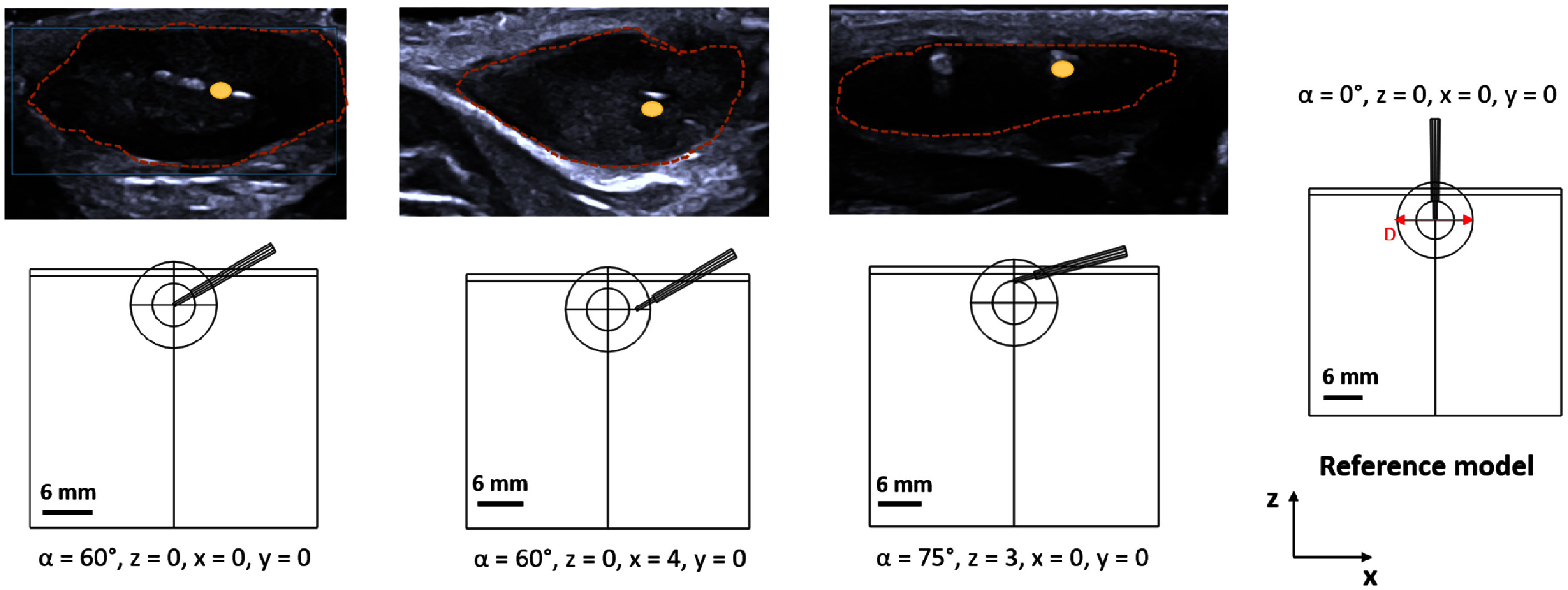
(first line) Variations in the RF-applicator position observed in B-mode ultrasound imaging. (Second line) Computational models of the three most recurrent scenarios to account for the inclination angle (α) and variabilities in the position (x, y, z) of the RF-applicator relative to the center of the tumor geometry estimated from B-mode ultrasound imaging against the reference scenario of RF-applicator centrally positioned and perpendicular to the tumor surface. The yellow marker on B-mode images indicates the position of the RF-applicator tip within the tumor. The red line is an approximation of the tumor boundaries.

We identified three recurrent scenarios of the position of the needle as the example ultrasound images show in figure 2.

The position of the RF-needle of the reference geometry was modified to represent each of the three experimental scenarios, where the RF-needle position is not perpendicular to the surface (*α* ≠ 0°) and:

• approximately at the center of the tumor (*α* = 60°, *x* = *y* = *z* = 0 mm)
• shifted toward the periphery of the tumor at the interface with the muscle (*α* = 60°, *x* = 4 mm, *y* = *z* = 0 mm);
• shifted toward the skin surface (*α* = 75°, *x* = *y* = 0 mm, *z* = 3 mm).

*3. Maximum diameter and shape of the tumor*

In the experiments we conducted, the size of each tumor was measured both with calipers before starting each treatment and via ultrasound imaging. The average value of the maximum tumor diameter (*D* = 12 mm) was consistent with the value reported from previous preclinical studies (De Vleeschauwer *et al* 2024) and consistent with the diameter of the spherical geometry we used in the reference model scenario (figure 1). However, tumor maximum diameter varied considerably across all treated tumors, with *D* ranging between 7 mm and 21 mm. Ultrasound imaging also showed relevant differences in the shape of tumors especially between tumors of small (∼7–10 mm) and large maximal diameter (>15 mm): smaller tumors tended to be approximately spherical, whilst the shape of larger tumors could be either spherical or irregular. In order to study how these differences in the circularity and size of the tumors may influence the temperature profiles, we modified the tumor geometry of the reference scenario (figure 1) to model three representative experimental scenarios:

• large tumors, approximately circular (*D* = 20 mm and *d*/*D* = 0.8);
• small tumors, approximately circular (*D* = 7 mm and *d*/*D* = 0.8);
• large tumors of irregular shape (*D* = 20 mm and *d*/*D* = 0.3).

The dimensions are relative to the maximum diameter (*D*) of the geometry. The changes in the tumor circularity are indicated by the ratio between minor axis diameter (*d*) and major axis diameter (*D*).

*4. Arterial flow of the major blood vessel within the tumor*

The reference scenario model was built without accounting for the presence of major blood vessels within the tumor volume. Doppler ultrasound imaging applied before RFHT showed the presence of a relatively large vessel within most of the treated tumors. The high blood velocity within large arteries is the cause of major heat sink that could significantly impact the thermal profile (Haemmerich *et al* 2003b, Liu *et al* 2006, Mohammadi *et al* 2022).

**Figure 3.**
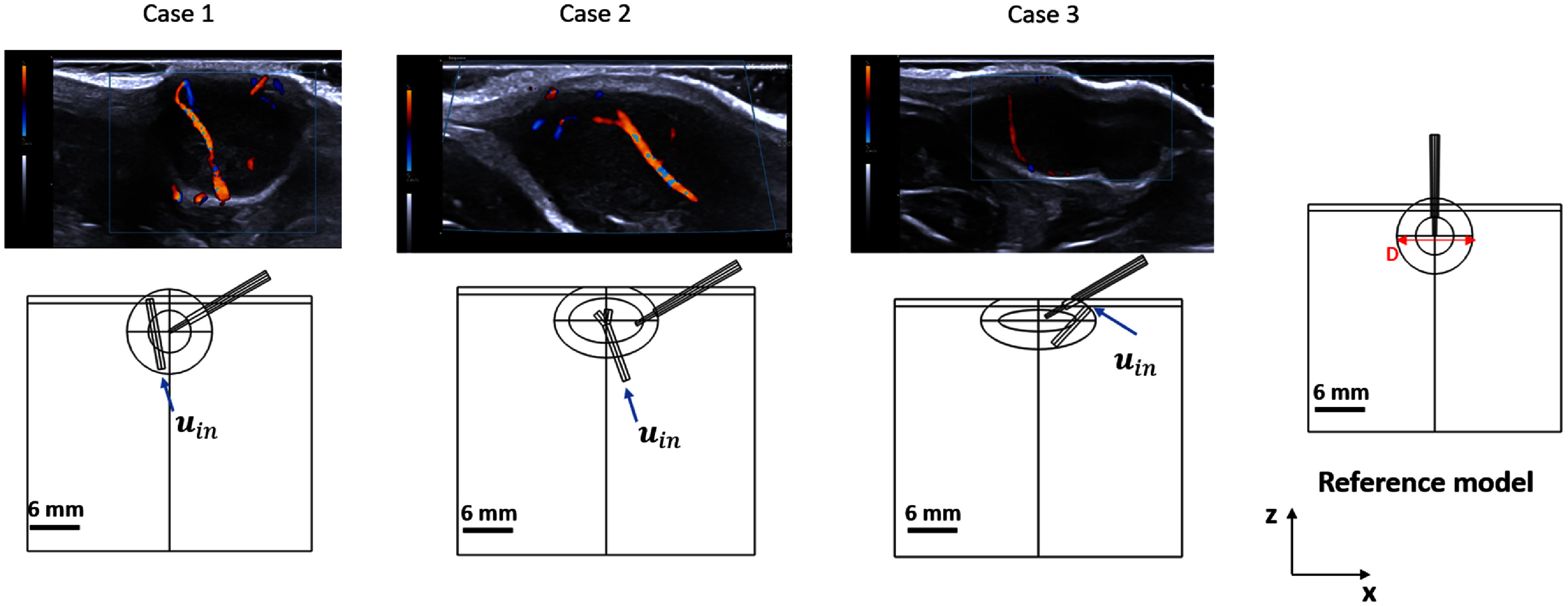
(First line) Variations in the location of a major blood vessel within tumors of different diameter and shape identified from Doppler ultrasound imaging. (Second line) Computational models of three experimental case scenarios, including realistic values of blood velocity and direction of the flow and B-mode ultrasound imaging information of the anatomical characteristics of the tumor and RF-applicator position, compared against the no-vessel reference scenario.

We conducted a qualitative analysis of the available recordings of Doppler ultrasound images and selected three experimental case scenarios as shown in figure 3. The selected three scenarios are visibly different in terms of the geometry and position of the vessel relative to the center of the tumor, tumor diameter and circularity, direction of the blood flow, indicated by the position of the draining vein (blue) and the artery (red).

Thus, we built the corresponding representative computational models. The geometrical parameters and corresponding value/description that were modified in the reference scenario model to account for the presence of the blood vessel are listed in table 2 for each case scenario. Where bifurcations in the vessels were noticed from Doppler imaging (Case 2), a symmetric bifurcation geometry was modeled in order to account for changes in the blood flow pattern such as those induced by blood recirculation that could further influence the temperature distribution within the adjacent tissue as compared to the straight geometry of the blood vessel (Case 1 and Case 3) (Liu *et al* 2006).

**Table 2.** Geometrical parameters of the tumor (i.e. maxium diamater, tumor circularity), needle position (x,y,z) and inclination (α), blood vessel approximated length and location within the tumor informed by B-mode ultrasound images and Doppler ultrasound recordings.

Case no.	Max. tumor diameter (*D*)	Tumor circularity (*d*/*D*)	Needle position (*α, x, y, z*)	Blood vessel location	Blood vessel length
1	12 mm	0.8	60°, *x* =*y* =*z* =0 mm	∼central	10 mm
2	14 mm	0.6	60°, x = 5 mm, *y* =*z* =0 mm	∼lateral	8 mm
3	16 mm	0.4	60°, *x* =*y* =*z* =0 mm	∼central	7 mm

The diameter of the blood vessel was assumed constant among the three case scenarios and equal to 1 mm in accordance to the size of medium caliber blood vessels in small animals (Swartz and Andreadis 2013).

The bioheat transfer equation (equation (1)) was modified to include the term of convection ($\rho c{\boldsymbol{u}} \cdot \nabla T$) relative to the blood flow within a major vessel (equation (2)).

\begin{equation*}\rho c{\text{ }}\frac{{\partial T}}{{\partial t}} = \nabla \cdot k\left( T \right)\nabla T + {{Q}}\left( {{T}} \right) - {\text{ }}{c_{\text{bl}}}{m_{\text{bl}}}\left( {T - {T_{\text{bl}}}} \right) - {\text{ }}\rho c{\boldsymbol{u}} \cdot \nabla T\end{equation*}

where ${\boldsymbol{u}}$ vector indicates the blood velocity. The fluid velocity profile within the blood vessel domain was solved combining the momentum (equation (3)) and mass conservation equations (equation (4)), under the assumption of incompressible ($\rho \nabla \cdot {\boldsymbol{u}}$ = 0), homogenous flow ($\frac{{\partial \rho }}{{\partial t}} = 0$) and negligible volume forces (${\boldsymbol{F}} = 0$) (González-Suárez *et al* 2018, Mohammadi *et al* 2022).

\begin{align*}{\rho _{\text{bl}}}\frac{{\partial {\boldsymbol{u}}}}{{\partial t}} = - \nabla P + {\text{ }}\mu {\nabla ^2}{\boldsymbol{u}} + {\boldsymbol{F}}\end{align*}

\begin{align*}\frac{{\partial {\rho _{\text{bl}}}}}{{\partial t}} = {\text{ }}{\rho _{\text{bl}}}{\text{ }}\left( {\boldsymbol{u} \cdot \nabla } \right){\boldsymbol{u}}\end{align*}

where, $ - \nabla P$ indicates the difference of pressure ($P$) between artery and vein, *μ* is the viscosity of the blood (table 1) and $\mu {\nabla ^2}{\boldsymbol{u}}$ the term indicating viscous forces during the motion of blood within the vessel. The steady state of the laminar flow remained fully developed for 15 min electromagnetic-bioheat transfer simulation, thus the flow within the vessel domain was independent of the heat transfer within the tissue.

Boundary conditions of the fluid-dynamic model are indicated by input velocity (${\boldsymbol{u}_{\text{in}}}$) applied to the surface of the blood vessel domain (figure 3) in each model, to account for the direction of the blood flow consistent with the corresponding Doppler images. Experimental assessments of blood velocity from Doppler imaging were in the range 1.5 cm s^−1^–2.5 cm s^−1^, thus we used a representative value of 2 cm s^−1^ in simulations for cases 1–3. Further analysis indicated a maximum discrepancy of 0.5% in *V*_50_ and 1.0% in *V*_43_ when using the representative averaged value for blood velocity, rather than actual case-specific values. The averaged value of 2 cm s^−1^ was used as the input velocity boundary condition (${\boldsymbol{u}_{\text{in}}}$ = 2 cm s^−1^) for all three simulated case scenarios. Zero value pressure (0 Pa) was applied to the opposite surface of the vessel geometry to model the draining vein. In the case of the bifurcated geometry, boundary conditions of 0 Pa were applied to the surface of both branches of the vessel geometry.

### 2.2. Metrics for the qualitative and quantitative assessment of thermal profiles

Spatial temperature profiles at the end (15 min) of low and high RFHT for all modeled scenarios are provided in both frontal (*zx-plane*) and axial planes (*xy-plane*) with reference to the axis of the RF applicator. In each temperature map, the extent of the 43 °C isotherm was used to qualitatively estimate the extent of the temperature gradient that can induce irreversible damage to cancer cells and blood vasculature over 15 min (Dewhirst *et al* 2022). We used ${V_{50}} = {\text{ }}\frac{{V_{T \unicode{x2A7E} 50^\circ C}^{\text{tumor}}}}{{{V^{\text{tumor}}}}} \cdot 100$ and ${V_{43}} = {\text{ }}\frac{{V_{T &lt; {\text{ }}43^\circ C}^{\text{tumor}}}}{{{V^{\text{tumor}}}}} \cdot 100$ as metrics for quantifying the percentage of the tumor volume where temperature is higher than 50 °C and lower than 43 °C, respectively, after delivering a selected thermal dose. The use of isotherms to quantify *V*_43_ and *V*_50_ is suitable for this study, given than the duration of RFHT (i.e. 15 min.) was the same for all treated tumors. For studies where varying duration thermal exposures are considered, use of metrics that employ time-temperature history are warranted (Pearce 2010).

The ${V_{50}}$ (‘ablation thermal volume’) metric provides quantitative information on the estimated fraction of thermal coagulation necrosis of the tumor volume which typically occurs when temperatures exceed 50 °C–60 °C. Similarly, ${V_{43}}$ (‘non-lethal thermal volume’) is used to estimate the fraction of the tumor volume where the blood microvasculature and blood flow is likely to remain intact (i.e. no stasis) at the end of the thermal intervention. This metric is relevant when subsequent treatments requiring systemic injection of the therapeutic agents are planned. Indeed, to ensure the effective delivery and distribution of any drug within the tumor, it is critical to preserve the blood supply.

This study does not consider the impact of thermal latency (i.e. impact of accumulated heat after power is terminated) as the metrics for assessing lethal and sub-lethal zones are based on isotherms. We note that prior studies (Ewertowska *et al* 2018) have demonstrated negligible impact of thermal latency on the ablation zone for long duration thermal exposures, such as the 15 min heating period considered in the present study. If considering the sub-lethal zone based on time-temperature considerations, thermal latency may have an impact, however this is anticipated to be similar for treatments of similar duration.

## 3. Results

${V_{50}}$ and ${V_{43}}$ values are reported in table 3 for all simulated scenarios and both thermal doses used in our previous experimental work (Bottiglieri *et al* 2025). In the following parts of the manuscript, we will focus the description and the discussion of the results related to the high RFHT only, which is the most clinically relevant thermal dose for RF thermal ablation procedures; all results about the low RFHT are provided in the Supplementary materials.

**Table 3.** Percentage values of the tumor volume fraction above 50 °C (V_50_) and below 43 °C (V_43_) assessed at the end (15 min) of high and low RFHT electromagnetic-heat transfer simulations for each value/case scenario of boundary conditions, RF-needle position, tumor geometry and blood vessel location within the tumor (i.e. Case 1, Case 2 and Case 3).

Variable	Description/value	High RFHT		Low RFHT	
		^a^${V_{50}}$(%)	^b^${V_{43}}$(%)	${V_{50}}$ (%)	${V_{43}}$ (%)
Boundary conditions	*h* = 5 $\left( {{\text{W}}{{\text{m}}^{ - 2}}{{\text{K}}^{ - 1}}} \right)$	30.8	24.7	0.8	94.3
	*h* = 15 $\left( {{\text{W}}{{\text{m}}^{ - 2}}{{\text{K}}^{ - 1}}} \right)$	24.9	34.6	0.5	96.1
	*T* = const = 30 °C	9.6	73.6	0.2	97.8

RF-needle position	*α* = 60°, *x* =*y* =*z* =0 mm	17.2	51.6	0.4	97.3
	*α* = 60°, *x* =4 mm, *y* =*z* =0 mm	9.1	77.8	0.2	98.2
	*α* = 75°, *z* =3 mm, *x* =*y* =0 mm	11.9	69.2	0	98.8

*D* (mm), *d*/*D*	20 mm, 0.85	4.8	85.4	0.06	99.2
	7 mm, 0.86	57.5	3.7	0.1	90.9
	20 mm, 0.3	15.2	55.3	0.02	98.5

Blood vessel position	Case 1	12.5	65.2	0.2	98.1
	Case 2	13.2	74.2	0.2	97.5
	Case 3	18.8	58.2	0.01	97.9

Reference scenario		19.0	49.4	0.3	97.4

^a^ ${V_{50}} = {\text{ }}\frac{{V_{T \unicode{x2A7E} 50^\circ C}^{\text{tumor}}}}{{{V^{\text{tumor}}}}} \cdot 100$.

^b^ ${V_{43}} = {\text{ }}\frac{{V_{T &lt; {\text{ }}43^\circ C}^{\text{tumor}}}}{{{V^{\text{tumor}}}}} \cdot 100$.

Table 3 shows *that the overall range of variability when considering all factors studied is 4.8%–57.5% for V_50_ and 3.7 %–85.4% for V_43_.* Compared to the reference scenario of a generic tumor geometry, with no blood vessel and the RF-applicator positioned perpendicular to the surface of the tumor, the absolute differences in *V*_50_ and *V*_43_ considering variabilities in (1) boundary conditions, (2) RF-needle position, (3) Size (*D*) and shape (*d*/*D*) of the tumor and (4) Blood vessel position, are: (1) 5.9%–11.8% (*V*_50_), 14.8 %–24.2% (*V*_43_); (2) 2%–10% (*V*_50_), 2%–28% (*V*_43_); (3) 4%–38% (*V*_50_), 6%–46% (*V*_43_); (4) 0.2%–6% (*V*_50_), 10%–25% (*V*_43_).

*1. Effect of ultrasound gel on the skin in the subcutaneous tumor model*

**Figure 4.**
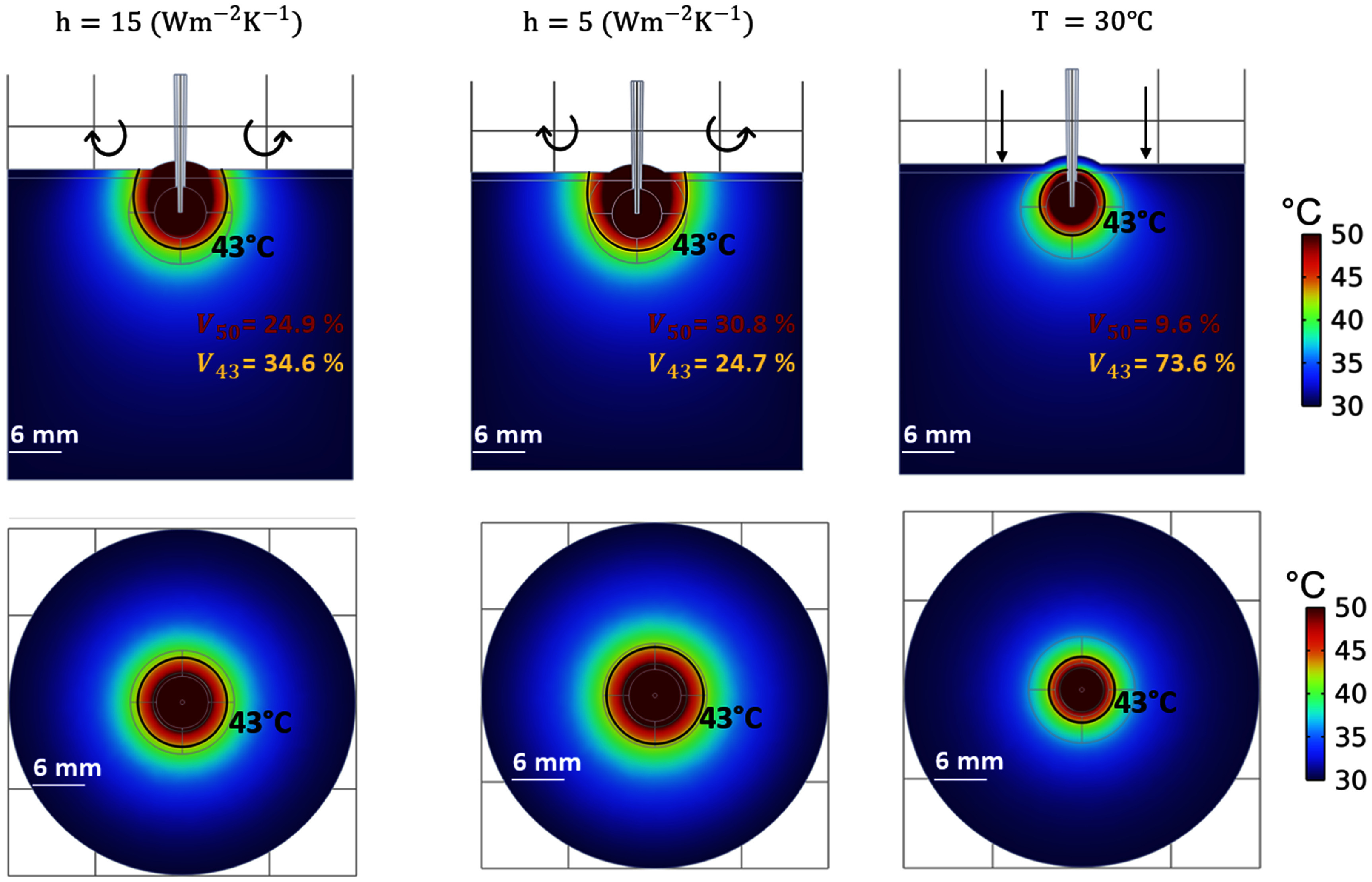
Simulated temperature profiles in the frontal (first line) and transverse planes (second line) at 15 min of high RFHT in the case of (1) *h* = 15 $\left( {{\text{W}}{{\text{m}}^{ - 2}}{{\text{K}}^{ - 1}}} \right)$, (2) *h* = 5 $\left( {{\text{W}}{{\text{m}}^{ - 2}}{{\text{K}}^{ - 1}}} \right)$ (3) *T* = 30 °C boundary conditions assigned at the outer surface of the tumor geometry (skin layer) to assess the influence of the ultrasound gel applied to the animal skin for ultrasound imaging-guidance of the RF applicator.

Figure 4 shows the effect of reduced heat exchange as modeled by applying boundary conditions of *h* = 15 (${\text{W }}{{\text{K}}^{ - 1}}{{\text{m}}^{ - 2}}$), *h* = 5 (${\text{W }}{{\text{K}}^{ - 1}}{{\text{m}}^{ - 2}}$), or by fixing the temperature at 30 °C at the external surface of the skin layer model. The surface of the skin visibly exceeds 50 °C both in the case of *h* = 15 (${\text{W }}{{\text{K}}^{ - 1}}{{\text{m}}^{ - 2}}$) and *h* = 5 (${\text{W }}{{\text{K}}^{ - 1}}{{\text{m}}^{ - 2}}$), caused by the limited removal of the heat from the circulation of the air. A contrasting effect on the temperature profile is visible in the scenario of the constant *T* = 30 °C boundary condition, where the increase of the temperature within the tumor region closer to the surface is below 43 °C.

The changes that have been qualitatively estimated within the tumor domain in proximity of the skin surface reflects the quantitative analysis of the percentage of the tumor volume where temperature is above 50 °C (${V_{50}}$) or below 43 °C (${V_{43}}$). ${V_{50}}$ and ${V_{43}}$ are between ∼9%–31% and ∼24%–74% depending on the applied boundary conditions (table 3). In particular, *T* = 30 °C and *h* = 5 $\left( {{\text{W }}{{\text{K}}^{ - 1}}{{\text{m}}^{ - 2}}} \right)$, yield the highest differences in the temperature profile as compared to the reference model. A decrease of 49% (*T* = 30 °C) and an increase of 62% (*h* = 5 $\left( {{\text{W }}{{\text{K}}^{ - 1}}{{\text{m}}^{ - 2}}} \right)$) in ${V_{50}}$ are found as compared to the reference model scenario. In turn, the volume of tumor tissue that is estimated to be below the lethal temperatures (${V_{43}}$) shows a difference of ∼50 %, increasing in the case of *T* = 30 °C and decreasing in the case of *h* = 5 $\left( {{\text{W }}{{\text{K}}^{ - 1}}{{\text{m}}^{ - 2}}} \right)$, compared to the reference. These results point to differences in thermal profiles that can be anticipated in studies where ultrasound imaging (and thus coupling gel) is used, compared to those where ultrasound imaging is not used.

*2. Effect of the position of the RF-applicator*

**Figure 5.**
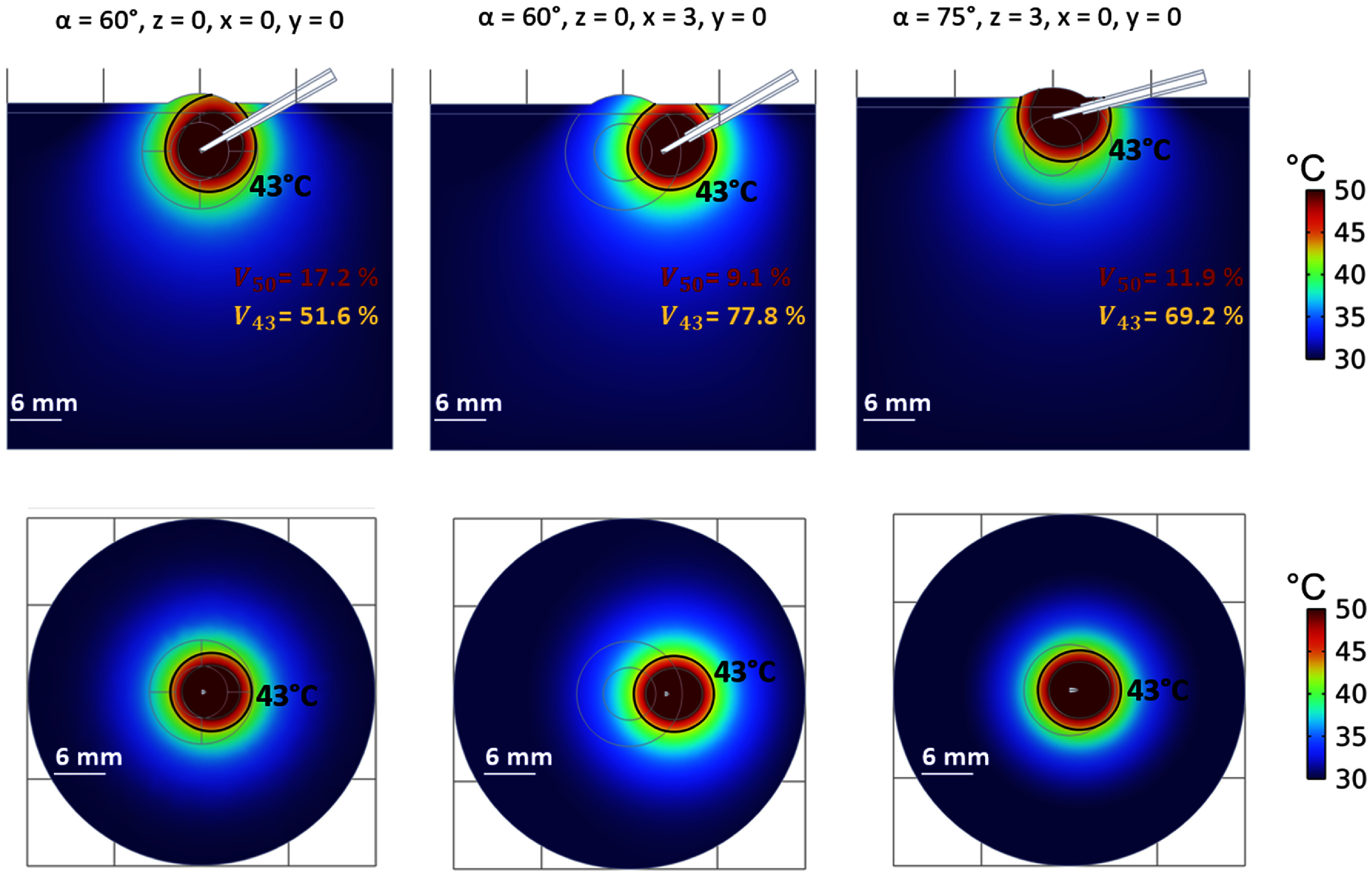
Simulated temperature profiles in the frontal (first line) and transverse planes (second line) at 15 min of high RFHT in three cases representative of (1) inclined RF-applicator and centrally positioned within the tumor, (2) inclined RF-applicator and positioned at the periphery of the tumor abutting the muscle; (3) inclined RF-applicator and positioned at the periphery of the tumor abutting the skin.

The spatial distribution of the temperature resulting from 15 min of high RFHT are presented in figure 5 as a function of the position of the RF-applicator within the tumor. Qualitative analysis of the temperature maps shows that the temperature gradient might involve or exclude, different regions of the tumor depending on the position of the ablation applicator. It can also be observed that the shaping of the temperature gradient is influenced by the proximity of the RF-applicator to the boundaries with tissues/materials of different electrical and thermal properties compared to those of the tumor tissue.

Values of ${V_{50}}$ and ${V_{43}}$ (table 3) in the case of RF-applicator remained approximately in the central area of the tumor (figure 5, *left*) are similar to those of the reference model scenario where a perpendicular alignment of the RF-applicator to the surface of the tumor geometry was assumed. In both cases, less than 20% (17.2%–19.0%) of the tumor volume is expected to be exposed to ablative temperatures (*V*_50_) and approximately 50% (51.6%–49.4%) of the tumor tissue is expected to remain below the irreversible damage threshold (${V_{43}}$).

This scenario is likely to change when the RF-applicator is shifted toward the periphery of tumor either toward the adjacent muscle or the skin (i.e. upper surface of the tumor geometry). In both cases (figure 5 *middle* and figure 5 *right*), the percentage of tumor volume above 50 °C and below 43 °C thresholds are within 9%–12% (${V_{50}}$) and 69%–78% (${V_{43}}$). Thus, compared to the case of a centrally located needle, ablated tumor volumes decrease by 37%–52% (${V_{50}}$) and tumor volumes spared from lethal thermal effects increase by 40%–57% (${V_{43}}$) when the RF-applicator is shifted toward tissues adjacent to the tumor.

The results indicate that inadvertent and unpredictable changes in the position of the applicator, that may routinely occur across experiments, might expose off-target and normal tissues to temperatures above 50 °C, thus denaturing the biophysical and immunological environment of the normal tissue.

*3. Effect of the diameter and shape of the tumor*

**Figure 6.**
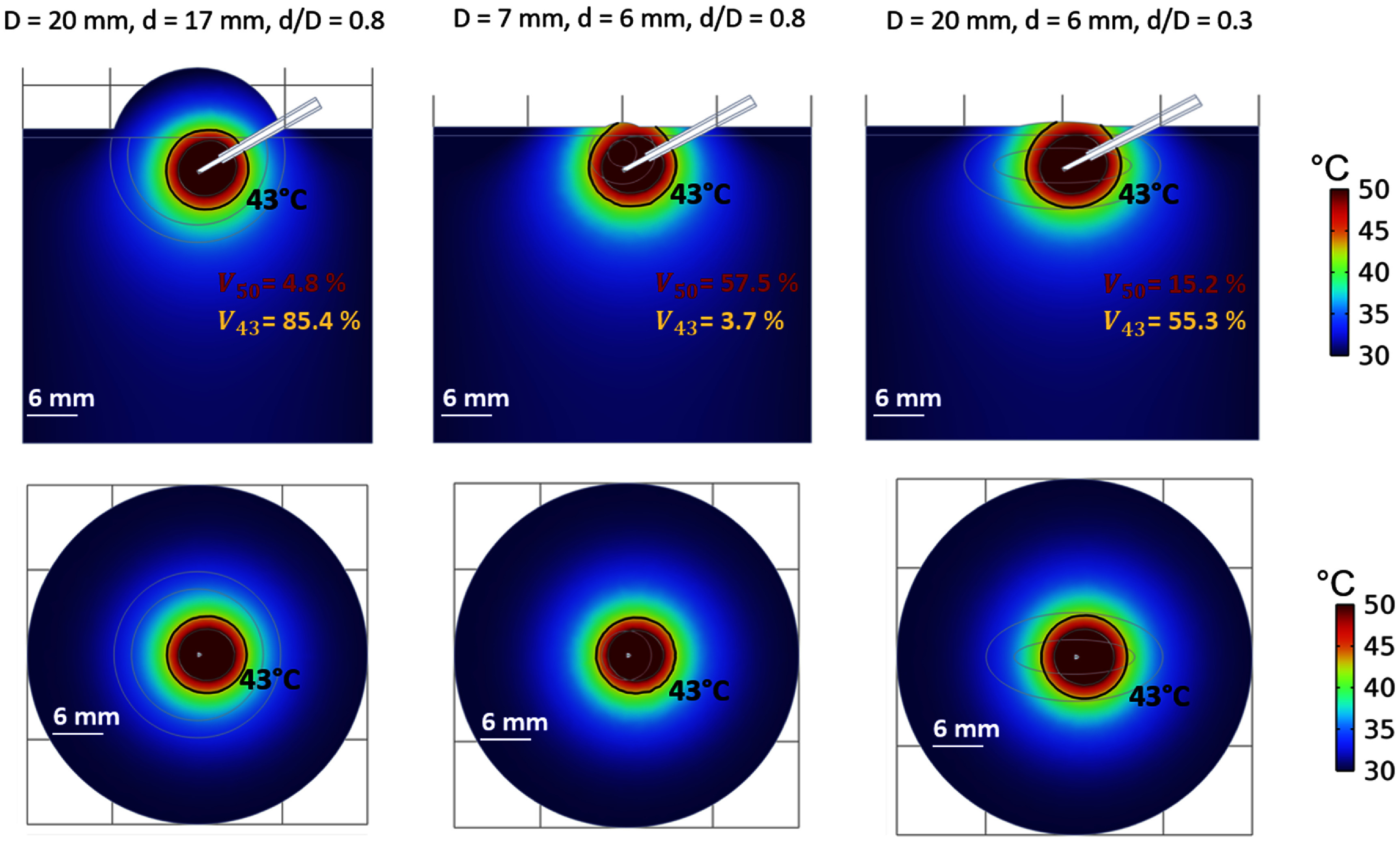
Simulated temperature profiles in the frontal plane (first line) and transverse planes (second line) at 15 min of high RFHT in three cases representative of (1) large (20 mm) tumor of approximately circular shape (d/D = 0.8), (2) small (7 mm) tumor of approximately circular shape (d/D = 0.8), (3) large (20 mm) tumor of non-circular/irregular shape (d/D = 0.3).

Figure 6 shows the effect of the variability in the maximum diameter (D) and circularity (d/D) among tumors on the spatial temperature profiles at 15 min of the simulated high RFHT. While the thermal profile remains similar across the three scenarios, how it aligns with the tumor/normal tissue geometry varies considerably across the cases. The quantitative analysis reveals a high variability in ${V_{43}}$ and ${V_{50}}$ (table 3) depending on the maximum diameter and circularity of the tumor. In relatively large and spherical tumors (*D* = 20 mm, *d*/*D* = 0.8), a decrease of more than 70% in ${V_{50}}$ is observed compared to the average size (*D* = 12 mm) assumed for the reference model, thus the ablated volume relative to the whole tumor becomes almost negligible (${V_{50}}$ = 4.8%). Conversely, in relatively small and spherical tumors (*D* = 7 mm, *d*/*D* = 0.8), 57.5% of the volume is expected to be ablated (${V_{50}}$) with 3.7% of tumor volume beneath the irreversible damage threshold (${V_{43}}$) as compared to 19.0% (${V_{50}}$) and 49.4% (${V_{43}}$) reported in the reference model.

The percentage of ablation volume (${V_{50}}$) can also change between tumors relatively similar in terms of maximum diameter, but different in terms of circularity, when treated with consistent thermal doses. In tumors with low circularity (*D* = 20 mm, *d*/*D* = 0.3) ${V_{50}}$ can yield an up to 3-folds increase (${V_{50}}$ = 15.2%) of the corresponding value (${V_{50}}$ = 4.8%) in tumors of similar maximum diameter, but of approximately circular shape (*D* = 20 mm, *d*/*D* = 0.8). As a consequence, the volume of tumor spared from irreversible effects of heating (${V_{43}}$) could become 0.6-folds smaller when the tumor assumes irregular shapes.

These results indicate that anatomical characteristics of the tumor should be reported not only in terms of average value of the maximum diameter or volume; the range of variability of the maximum size and information of irregular shapes should also be included.

*4. Effect of major intra-tumoral blood vessels*

**Figure 7.**
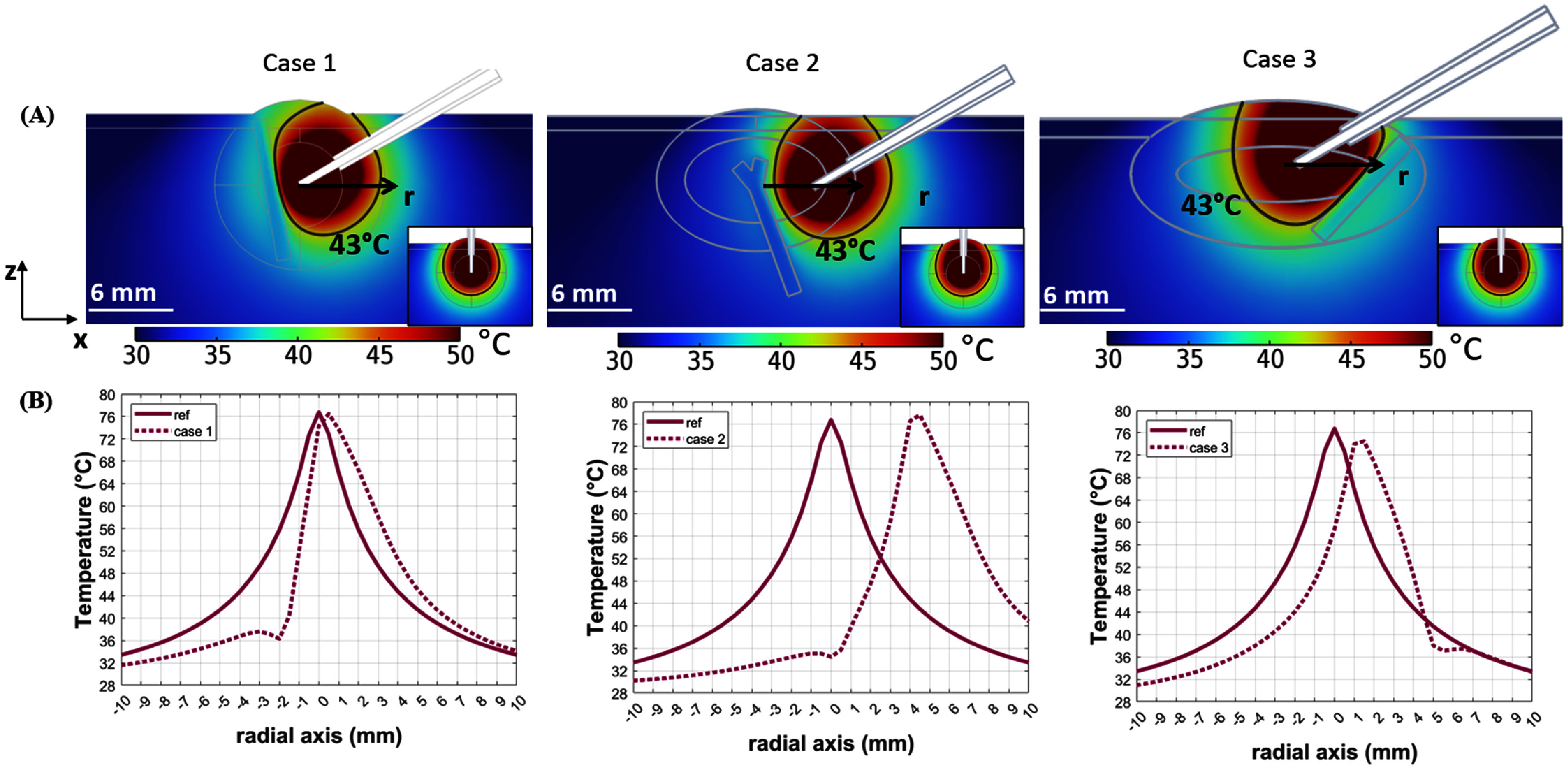
(first line) Simulated temperature profiles in the frontal plane at 15 min of high RFHT in three cases scenarios where the presence of a major blood vessel was considered: (1) major artery centrally positioned within the tumor and at a distance of 1.5 mm from the tip of the RF-applicator, (2) bifurcated artery at a distance of 4 mm from RF-applicator shifted to the periphery of the tumor abutting the muscle, (3) major artery located at the periphery of the tumor at a distance of 3 mm from the tip of a centrally positioned RF-applicator. (second line) Radial decay of the temperature from the tip of the RF-applicator in the three case scenarios of blood vessel positions against the symmetrical decay of the temperature in the case of no-vessel reference scenario.

Figure 7 (A) shows the temperature profile in the frontal plane (results in the transverse plane are provided in the Supplementary Material) relative to the long axis of the RF-needle in three computational models, representative of three experimental scenarios of different location and geometry of a major blood vessel within the tumor. The 2D spatial temperature profile is shown at 15 min of heating against the spatial temperature profile of the reference model scenario (right-corner quadrant figure 7). In contrast to the reference scenario of no-blood vessel and perpendicular position of the RF-applicator to the surface of the tumor geometry, in all three cases the blood vessel creates a physical barrier to the local heating, that in turn influences the final temperature gradient. The differences in and V43 (table 3) relative to the V50 and V43 of the reference scenario are –34% and 32% (Case 1), –30% and 50% (Case 2) and –1% and 18% (Case 3). It is worthwhile noting that in Case 1 and Case 3, where the RF-applicator was similarly centrally located and similar to the reference scenario, 65.2% and 58.2% of the tumor volume remains below the 43 °C threshold highlighting the significance of assessing the influence of a major blood vessel when local heating is delivered in the 12–16 mm diameter tumors typical of preclinical experiments.

The heat sink effect caused by the blood vessel location specific to each case scenario is highlighted in figure 7(B). The radial decrease of the peak temperature in both positive $\left( {{{\left. {\frac{{\partial T}}{{\partial r}}} \right|}_{{r^ + }}}} \right)$ and negative $\left( {{{\left. {\frac{{\partial T}}{{\partial r}}} \right|}_{{r^ - }}}} \right)$ radial direction was calculated at steps of 1 mm from the needle-tip position in each case scenario and the values are reported in table 4.

**Table 4.** Values of the temperature gradient along the positive (r^+^) and negative (r^–^) radial axis from the position of the RF-applicator in three case scenarios of blood vessel positions within the tumor at distances from the RF-applicator of 1.5 mm, 4 mm and 3 mm.

Case scenario	RF-applicator −1 mm		2–3 mm		4–5 mm		6–7 mm		8–9 mm	
	${\left. {\frac{{\partial T}}{{\partial r}}} \right|_{{r^ + }}}$	${\left. {\frac{{\partial T}}{{\partial r}}} \right|_{{r^ - }}}$	${\left. {\frac{{\partial T}}{{\partial r}}} \right|_{{r^ + }}}$	${\left. {\frac{{\partial T}}{{\partial r}}} \right|_{{r^ - }}}$	${\left. {\frac{{\partial T}}{{\partial r}}} \right|_{{r^ + }}}$	${\left. {\frac{{\partial T}}{{\partial r}}} \right|_{{r^ - }}}$	${\left. {\frac{{\partial T}}{{\partial r}}} \right|_{{r^ + }}}$	${\left. {\frac{{\partial T}}{{\partial r}}} \right|_{{r^ - }}}$	${\left. {\frac{{\partial T}}{{\partial r}}} \right|_{{r^ + }}}$	${\left. {\frac{{\partial T}}{{\partial r}}} \right|_{{r^ - }}}$
Case 1	−6.2	**−13.2**	−8.3	**−3.3**	−4.4	−1.1	−2.2	−1.0	−1.3	−0.7
Case 2	−7.5	−10.5	−8.4	**−8.8**	−4.3	**−0.6**	−2.2	−0.8	−1.3	−0.7
Case 3	−8.1	−8.4	**−12.6**	−7.0	**0.3**	−3.0	−1.3	−1.7	−1.0	−1.1
Reference	−10.8	−10.9	−6.5	−6.5	−3.3	−3.2	−1.9	−1.9	−1.2	−1.2

Figure 7(B) shows the contrast between the reference scenario, where the temperature decays symmetrically from the central position (*r* = 0 mm) of the RF-needle tip along the radial axis, and all three cases that present with asymmetrical temperature decays. In the selected case scenarios, a clear heat-sink is visible at distances of 1.5 mm from the applicator (Case 1), 4 mm (Case 2) and 3 mm (Case 3) and the transmural temperatures, i.e. the temperature crossing the blood vessel wall, remains below 40 °C.

However, the magnitude of the heat sink depends on the distance between the blood vessel and the applicator, thus it differs among the three cases as the variability in the values of the temperature gradient reported in table 4 suggests. The temperature difference between the two opposing sides $\left( {\frac{{\partial T}}{{\partial r}}{|_{{r^ + }}} - {\text{ }}\frac{{\partial T}}{{\partial r}}{|_{{r^ - }}}} \right)$ caused by the blood vessel reaches a maximum absolute value of 7 °C (Case 1), 3.7 °C (Case 2), and 5.6 °C (Case 3). These different values occur between RF applicator −1 mm (Case 1), 4–5 mm (Case 2) and 2–3 mm (Case 3) which are the regions where the blood vessel of the corresponding scenario is located. At the crossing of the blood vessel, the temperature gradient sharply changes from −13.2 to −3.3 °C mm^−1^ in Case 1, from −8.8 to −0.6 °C mm^−1^ in Case 2, from −12.6–0.3 °C mm^−1^ in Case 3 (highlights table 4). The quantitative analysis of the temperature gradient for each case aligns with the qualitative assessment of figure 7 that shows two distinct thermal regions caused by the presence of the blood vessel with transmural temperatures approaching physiological conditions (*T* < 40 °C) for all three case scenarios.

These results indicate that a blood vessel of relatively large caliber, that in small animals ranges between 1$ - $2 mm (Swartz and Andreadis 2013), could pose a substantial physical barrier to the heat distribution and thermally spare relevant portions of the tumor volume from the levels at which bioeffects can be induced. A quantitative analysis of ablated vs unablated volume as reported in table 3 coupled with the analysis of the temperature gradient in relation to the position of the RF-heating applicator could expand the understanding of the distributions of lethal (*T* > 50 °C), sublethal (40 °C ⩽ *T* < 43 °C) and non-lethal (*T* < 40 °C) temperatures within the tumor and the potential involvement of adjacent normal tissues.

## 4. Discussion

Several studies primarily conducted in preclinical settings of small animal tumor models showed mixed results on how local thermal interventions could be considered in synergistic approaches with other therapies. The temporary increase of the blood perfusion and decrease of intratumoral pressure driven by the tumor regions exposed to hyperthermia temperature ranges, might facilitate the delivery and the distribution of drugs within the tumor (Dewhirst and Secomb 2017, Izci *et al* 2021). However, the heating to higher temperatures in most of the tumor volume might exacerbate the stiffness of the extracellular matrix (Odéen *et al* 2023) and stymie the distribution of therapeutic agents. Local thermal interventions also affect the tumor microenvironment; increased infiltration of dendritic cells, T-cells, and NK cells that could potentiate the effect of immunotherapies as well as mechanisms that promote tumorigenesis, such as via HIF-α/VEGF, have been observed after local heating in small animal tumor models (Markezana *et al* 2020, 2021, Muñoz *et al* 2022, Santana *et al* 2023).

These heat-induced bioeffects are highly dependent on the temperature profile over the course of the thermal exposure period, which is far from being uniform within the tumor. Indeed, lethal temperatures (*T* > 50 °C) that induce coagulation necrosis of the tumor cells, sublethal temperatures (40 °C ⩽ *T* < 43 °C) that induce temporary increase of blood perfusion and permeability of blood vessels and non-lethal temperatures (*T* < 40 °C) are all part of the temperature gradient observed in local heating.

The percentage of the tumor volume exposed to different temperature ranges of the post-ablation temperature gradient influences the consequent bioeffects that ultimately could influence the treatment outcome. Characterizing and interpreting the time-temperature profile within tumors may be particularly important in the context of combination treatments, where the interplay between heat-induced bioeffects and other therapeutics may occur at temperatures below the ablative threshold (Tak *et al* 2018).

Despite the increased awareness that time-temperature dynamics is one of the determinants of the heat-induced biological effects, methods to deliver and assess temperature-based therapies are often oversimplified in preclinical studies of thermal ablation in small animal tumor models. In addition, the lack of characterization and reporting of the temperature profiles after each treatment, limits not only the reproducibility of the experimental studies but also the understanding of the heat-induced effects within the tumor and the interpretation of the subsequential biological and immunological responses.

In this study we report on the effect on the temperature profiles of parameters we identified as potential influencing factors during our previous experimental study, where a customized instrumentation system was used to deliver local RFHT within a rat model of HCC (Bottiglieri *et al* 2025).

We developed a computational bioheat transfer modeling approach to include available experimental information extracted from B-mode ultrasound imaging, Doppler ultrasound, and experimental reports for each tumor treated at the designed thermal dose. Then, we assessed the variation in the percentage of tumor volume within lethal (ablation) temperature range (${V_{50}}$) and below the irreversible damage temperature threshold (${V_{43}}$) at the end of delivering a consistent thermal dose (i.e. power > 1 W, for 15 min, peak temperature ∼77 °C, time-to-peak ∼3–4 min (Bottiglieri *et al* 2025)). As a function of: (1) convective heat exchange between skin and air caused by the application of ultrasound gel, (2) shifting in the position of the needle within the tumor, (3) diameter and circularity of the tumor, and (4) position of a major vessel within the tumor and related blood flow profile.

When considering together factors (2) through (4), the relative ablation volume estimated by ${V_{50}}$ can vary between ∼4%–58%. Similar findings were obtained for the relative non-lethal thermal volume estimated by (${V_{43}}$) which can vary between ∼3%–78% (table 3).

The assumption of a stable position of the applicator throughout each procedure is challenged by a number of experimental factors including anatomical characteristics of the tumor (e.g. size, shape, fraction of liquid vs solid components) and unpredictable experimental confounders. The heat-induced bioeffects are likely to be influenced by the electrical and thermal characteristics of the tissue where the needle shifted to and the subsequent local temperature increase. For example, this study shows that a shift of the needle position of 4 mm toward the boundary of the tumor, could decrease the tumor ablation volume (${V_{50}}$) by about 50% as compared to a centrally located needle scenario, and increase the risk of damaging adjacent normal tissues (figure 5). In addition, the increase of the extent of normal tissue exposed to ablation temperatures could elicit biological effects via pathways different than the cases where most of the ablation is confined within the tumor volume.

Differences in the diameter and shape of the tumor can further contribute to the variability in the temperature profile. Most preclinical studies report either the average of the largest dimension of the tumor or tumor volume. In this study we showed that despite the averaged diameter (∼12 mm) measured across the tumors included in the experimental study being consistent with other preclinical studies using a similar tumor model (Muñoz *et al* 2022, Tian *et al* 2022, Santana *et al* 2023, De Vleeschauwer *et al* 2024), a variability of about 50% of the average size was reported (7–20 mm). In addition, tumors of small diameter (<10 mm) tend to be more circular than those of larger dimension adding the tumor shape as a further influencing factor to the spatial temperature profile. Quantitative analysis of the diameter and shape of the tumor, assessed as independent variables, reveals that ${V_{50}}$ and ${V_{43}}$ can vary between 4%–58% and 3%–86%, respectively. In tumors of large diameter, most of the tumor volume is below lethal temperatures (${V_{43}}$ > 80%). In contrast, ablation temperatures are more likely to cover most of the volume (${V_{50}}$ > 55%) in tumors of relatively small dimension with a relatively small margin of tumor volume within non-lethal temperatures (<4%). These findings suggest that the estimates of the ablation zone relative to the tumor geometrical characteristics, either via computational models or histopathology (e.g. hematoxylin and eosin stained sections), should be included for the analysis of the heat-induced bioeffects in the cases of tumors presenting relevant differences in the diameter and shape as compared to the average within the same experimental group.

It is well known that the relatively high blood velocity within major blood vessels is among the main sources of variability in the extent of the ablation zone both in clinical and preclinical settings (Haemmerich *et al* 2003b, Abtin *et al* 2012). The growth of the ablation zone using RF energy is only in part governed by resistive (direct) heating, in fact it is mostly driven by thermal conduction pushing the heat toward peripheral areas of the tissue (Brace 2009). As a result, RF ablation is especially susceptible to local heat removal caused by the circulation of the blood, depending on the position of vessel relative to the RF applicator (Haemmerich *et al* 2003b). The heat sink effect caused by the proximity of major blood vessel could be even more prominent in small animal tumors, where ensuring adequate distance between the vessel and the applicator could be challenging. Using Doppler ultrasound imaging before the start of heating, we observed that the position of major blood vessels is different and unpredictable among tumors in the same experimental group. Thus, the 3D temperature profile at the end of even well-controlled heating could be highly affected by the distance of the large blood vessels from the position of RF-applicator. The present study shows that the ablated volume (${V_{50}}$) decreases by 1.1%–34.2%, depending on the vessel—RF-applicator distance, compared to the no-vessel reference model. Multiple factors including the distance between the major blood vessel and RF-applicator, tumor size, shape and shift of the RF-applicator toward adjacent tissues point to the overall impact of the heat sink on the temperature distribution within the tumor volume. Computational models of three experimental scenarios shows relevant differences in the temperature distributions (figure 7) with: cases (Case 1 and Case 3) where mostly central regions are exposed to temperatures higher than 43 °C and peripherical regions of the tumor below 40 °C (figure 7(B), table 4), cases (Case 2 and Case 3) indicating an increased risk of inducing coagulation necrosis of normal tissue, and cases (e.g. Case 2) where the tumor volume is largely left below 40 °C. Imaging-based assessment of the blood flow during the experiments proved instrumental for the development of computational models using physiological values specific to small animals (blood velocity ∼2 cm s^−1^) rather than relying on values reported across previous computational studies that refer to human physiology (blood velocity ∼50 cm s^−1^) (Haemmerich *et al* 2003b, Liu *et al* 2006).

Finally, when preclinical studies involving local heating are conducted in subcutaneous tumor models it is worthwhile to consider environmental parameters that can change the mechanism of heat exchange occurring at the surface of the skin and yield additional variability on the final temperature profile. In this study we considered the possible effect of the acoustic coupling gel, used for the ultrasound guided positioning of the RF applicator on the temperature profile at the end of the heating (figure 4). Based on the experimental evidence of a mild increase of the temperature caused by the viscosity of the gel but no sign of skin burns on the animals, we concluded that the initial assumption in our reference model provides a reasonable representation of the heat exchange mechanisms between skin and air. However, those boundary conditions might change with different heating techniques and laboratory conditions where the experimental study is conducted.

Overall, the findings of the present study demonstrate that a tumor-based analysis of the temperature distribution in preclinical investigations is relevant particularly in the cases of tumors presenting with a large blood vessel and with significant differences in dimension, shape and/or position of the RF-applicator within groups designed to yield similar baseline and experimental conditions. The experimentally-informed computational modeling approach presented here may provide the means to identify experimental scenarios where *in vivo* temperature profiles do not reflect the intended experimental designs. The approach presented in this study integrating experimental information and computational modeling may improve reliability when experimental groups are compared in terms of biophysical and immunological responses to combinatorial treatment approaches involving local thermal ablation.

In conclusion, the present study highlights that:

• reporting procedure-related parameters, including thermal dose, peak temperature values, time-to-peak temperature, and position of the heating applicator,
• identifying methods and metrics to quantitatively assess both temporal and spatial profiles of the temperature at the end of the heating,
• assessing the variability of tumor anatomical characteristics including tumor size, shape, position major blood vessels and related blood velocity,

are key aspects of preclinical studies designed to assess the biological and/or immunological ramifications of local thermal interventions. Taken together these aspects could allow for more reliable interpretation of heat-induced bioeffects, facilitate the reproducibility of the studies among different research groups, and advance the understanding of the potential risks and advantages of combined treatment approaches between systemic therapies and local-thermal interventions.

## Data Availability

All data that support the findings of this study are included within the article (and any supplementary information files).
